# Intracerebroventricular Leptin Infusion Improves Glucose Homeostasis in Lean Type 2 Diabetic MKR Mice via Hepatic Vagal and Non-Vagal Mechanisms

**DOI:** 10.1371/journal.pone.0017058

**Published:** 2011-02-17

**Authors:** Xiaosong Li, Xhiping Wu, Raul Camacho, Gary J. Schwartz, Derek LeRoith

**Affiliations:** 1 Departments of Medicine and Neuroscience, Albert Einstein College of Medicine, Bronx, New York, United States of America; 2 Department of Medicine, Mount Sinai School of Medicine, New York, New York, United States of America; Pennington Biomedical Research Center, United States of America

## Abstract

MKR mice, lacking insulin-like growth factor 1 receptor (IGF-1R) signaling in skeletal muscle, are lean yet hyperlipidemic, hyperinsulinemic, and hyperglycemic, with severe insulin resistance and elevated hepatic and skeletal muscle levels of triglycerides. We have previously shown that chronic peripheral administration of the adipokine leptin improves hepatic insulin sensitivity in these mice independently of its effects on food intake. As central leptin signaling has been implicated in the control of peripheral glucose homeostasis, here we examined the ability of central intracerebroventricular leptin administration to affect energy balance and peripheral glucose homeostasis in non-obese diabetic male MKR mice. Central leptin significantly reduced food intake, body weight gain and adiposity, as well as serum glucose, insulin, leptin, free fatty acid and triglyceride levels relative to ACSF treated controls. These reductions were accompanied by increased fat oxidation as measured by indirect calorimetry, as well as increased oxygen consumption. Central leptin also improved glucose tolerance and hepatic insulin sensitivity determined using the euglycemic-hyperinsulinemic clamps relative to pair fed vehicle treated controls, as well as increasing the rate of glucose disappearance. Hepatic vagotomy only partially reversed the ability of central leptin to improve glucose tolerance. These results demonstrate that central leptin dramatically improves insulin sensitivity independently of its effects on food intake, in a lean mouse model of type 2 diabetes. The findings also suggest that: 1) both hepatic vagal and non-vagal pathways contribute to this improvement, and 2) central leptin alters glucose disposal in skeletal muscle in this model.

## Introduction

MKR mice are a model of non-obese Type 2 diabetes created by engineering a muscle-specific dominant-negative insulin-like growth factor-1 receptor (IGF-1R) transgene that abrogates IGF-1R and insulin receptor (IR) function in skeletal muscle by forming hybrids exclusively with endogenous muscle IGF-1Rs [1]. The formation of similar hybrids with endogenous insulin receptors similarly inhibits their function by greater than 80%. As a consequence, MKR mice are born with severe muscle insulin resistance and subsequently develop insulin resistance in both liver and adipose tissue. Eventually β cell dysfunction occurs, as demonstrated by a loss of first phase insulin secretion, but with an exaggerated second phase insulin secretion. Thus, by 6–8 weeks of age, MKR mice characteristically demonstrate hyperinsulinemia, hyperglycemia, hyperlipidemia and increased tissue TG levels [2].

Results from our previous studies suggested that the rapid progression of MKR phenotype from pre-diabetic levels to frank diabetes is secondary to their hyperlipidemia and increased TG levels in muscle and liver. Accordingly, chronic peripheral administration of β adrenergic agonists or fibrates to MKR mice reduced tissue lipid levels and improved hepatic insulin sensitivity [3]. We subsequently found that chronic peripheral administration of the adipokine leptin to MKR mice also dramatically reduced adiposity, increased fat oxidation, and improved hepatic insulin sensitivity and glucose homeostasis, independently of its effects on food intake [4]. The sites and modes of leptin action responsible for these improvements remain unknown, but may involve the central nervous system; central leptin receptor signaling has been implicated in the control of multiple determinants of peripheral glucose homeostasis, including fatty acid oxidation, lipogenesis [5], hepatic insulin resistance [6], hepatic glucose fluxes [7], and skeletal muscle substrate utilization [8], [9]. Consequently, the present studies were designed to determine whether central leptin administration would be sufficient to recapitulate the beneficial metabolic effects of peripheral leptin in the lean Type 2 diabetic MKR mouse. As the hepatic vagus has also been implicated in the central leptinergic control of glucose homeostasis [10], we also examined whether the hepatic vagus mediated any effects of central leptin on glucose tolerance.

## Materials and Methods

### Animals

MKR Type 2 diabetic mice have been previously described [1]. Male MKR mice on an FVBn background were studied in these experiments. Homozygous MKR mice were identified by tail-vein fed glucose measurements ranging between 250–500 mg/dl, 6 to 8 weeks post-weaning, compared with WT mice with normal blood glucose levels (between 150–180 mg/dl). Throughout all studies, mice were maintained in a 12-h light/dark cycle and were fed standard NIH -07 diet with ad libitum access to tap water. All animal protocols were approved by the IACUC committees of the Mount Sinai School of Medicine and the Albert Einstein College of Medicine of Yeshiva University.

### Surgical Procedures

#### Third ventricle cannulation

Under i.p ketamine/xylazine anesthesia (Ketamine (100 mg/kg) and Xylazine (10 mg/kg) [11], 10–12 wk old MKR mice were stereotaxically implanted with a 28 G chronic stainless steel cannula (Plastics One Inc.) targeting the 3^rd^ ventricle (coordinates: A/P −1.6 mm posterior to bregma, D/V −4.7 mm). After a 1 week recovery period, mice were briefly anesthetized under 0.25% isoflurane, and an Alzet 1004 minipump (Cupertine, CA) was inserted subcutaneously on the back and connected to the 3^rd^ ventricle cannula via a polyethylene tubing connector kit (Alzet). Pumps were preloaded with either recombinant mouse leptin (R&D systems, Minneapolis, MN) dissolved in artificial cerebrospinal fluid (ACSF) or ACSF (Harvard Apparatus, Boston, MA). Infusion rate and duration was 0.25 ul/hour (leptin: 0.0417 ug/hour) for 5–14 days, depending on the study. Accurate 3rd ventricle cannula placement was confirmed by postmortem inspection of the cerebroventricular spread of a 500 nl India ink injection.

#### Hepatic branch vagotomy

Hepatic branch vagotomy was performed in Ketamine/Xylazine anesthetized animals as previously described [10]. Briefly, a laparotomy incision was made on the ventral midline and the abdominal muscle wall opened, revealing the gastrointestinal tract in the peritoneum. The gastrohepatic ligament was severed, and the stomach was gently retracted onto sterile saline soaked cotton gauze, revealing the descending ventral esophagus and the ventral subdiaphragmatic vagal trunk. The hepatic branch of this vagal trunk was visualized using a neurosurgical dissecting scope under 10–20× magnification, and the hepatic branch of the vagus was ligated using two 8.0 silk ties. The hepatic nerve trunk was then transected by microcautery between the two sutures, severing and cauterizing the hepatic vagus, thereby minimizing the possibility of regeneration. The muscle and skin layers of the laparotomy incision were closed with Prolene suture and surgical adhesive (Vetbond), respectively. After experiments, animals were sacrificed and the adequacy of the procedure was assessed by demonstrating the absence of vagal nerve tissue between the sutures. For sham surgeries, a midline laparotomy was performed and the hepatic vagal nerve trunk ware exposed but not manipulated or severed.

#### Vascular catheter implantation

One week prior to clamp studies, catheters were inserted in the right jugular vein (MRE-025, Braintree Scientific Inc.) and carotid artery (0007700, Alzet, Cupertino, CA) during anesthesia with 0.25% isoflurane.

### Energy Balance Phenotyping

For five days before and throughout a 14 day period of ICV leptin or ACSF infusion, animals were individually housed in metabolic chambers maintained at 21–22°C on a 12-hour light/12 dark cycle with lights on at 7 am, and were provided with nutritionally complete standard powdered diet (NIH 07) and tap water ad libitum. Metabolic and behavioral measurements (oxygen consumption, food intake, locomotor activity) were obtained continuously using a CLAMS (Columbus Instruments) open-circuit indirect calorimetry system. Body weights were measured daily, and body composition was determined weekly by magnetic resonance spectroscopy using an ECHOMRI instrument (Echo Medical Systems.). For pair fed groups, each animal received food in individual cages, and the amount of food provided was adjusted daily to correspond to the average amount of food consumed by the leptin treated group for each successive day of leptin treatement.

### Plasma Assessments

Serum insulin and leptin levels were measured using radioimmunoassay kits (Linco, St. Charles, MO). Serum free fatty acids and triglyceride levels were measured by enzymatic colorimetric assay (Roche, Penzberg, Germany and Thermo, Victoria, Australia, respectively). Blood glucose was measured by glucometer (Analox GM 7, Analox Instruments, Lunenburg, MA).

### Glucose Tolerance Tests

Two weeks following chronic 3^rd^ ventricle minipump implantation for leptin and ACSF control infusions, i.p. glucose tolerance tests (ipGTTs) were performed. Briefly, after a 5 hour fast, mice were injected intraperitoneally with glucose (2 g/kg BW) and blood glucose levels were determined from tail vein samples at 0, 15, 30, 60 and 120 minutes after the injection.

### Hyperinsulinemic-Euglycemic Clamp Studies

In separate studies designed to determine the mechanisms involved in the improvement in glucose homeostasis following 3^rd^ ventricle leptin infusion, hyperglycemic–euglycemic clamps were performed in unrestrained mice 5 days following the onset of the central minipump infusion. Vascular catheters were inserted as described above, 8 days prior to the clamps to allow for surgical recovery prior to minipump implantation. Mice were fasted for 5 h prior to clamps. The clamp protocol consisted of a 90-min tracer equilibration period (t =  −90 to 0 min) beginning at 12:00 P.M. followed by a 120-min experimental period (t = 0 to 120 min). A blood sample was obtained at t =  −90 min to determine initial glucose levels. A 5-uCi bolus of [3-3H] glucose purified by high-performance liquid chromatography was given at t =  −90 min followed by a 0.05 uCi/min infusion. At t = −15 and −5 min, a blood sample (10 ul) was taken for the assessment of basal glucose and insulin levels and glucose turnover. The insulin clamp was begun at t  = 0 min with a primed-continuous infusion of human insulin (300 mU/kg bolus followed by 3 mU/kg/min 1; Novolin; Novo Nordisk, Princeton, NJ). The [3-3H] glucose infusion was increased to 0.1 uCi/min for the remainder of the experiment. Euglycemia (120–130 mg/dl) was maintained during clamps by measuring blood glucose every 10 min starting at t = 0 min and infusing 45% dextrose as necessary. Blood samples (60–200 ul) were taken every 10 min from t = 80 to120 min and processed to determine glucose specific activity. Clamp insulin levels were determined from samples obtained at t = 80 and 120 min. All blood samples were resuspended in heparinized saline and infused back to mice to maintain hematocrit. Plasma samples were collected to determine glucose levels and specific activities of [3-3H]-glucose. All tissue samples were stored at −80°C for subsequent analysis.

### Statistical Analyses

Statistical comparisons were made by one way, two way or repeated measures ANOVAs, using Bonferroni correction for planned comparisons. An alpha level of 0<0.05 indicated significant differences among treatments. Unless otherwise specified, data are presented as means ± SEM.

## Results

### Food Intake, Body Weight And Locomotor Activity

**Figure 1:**
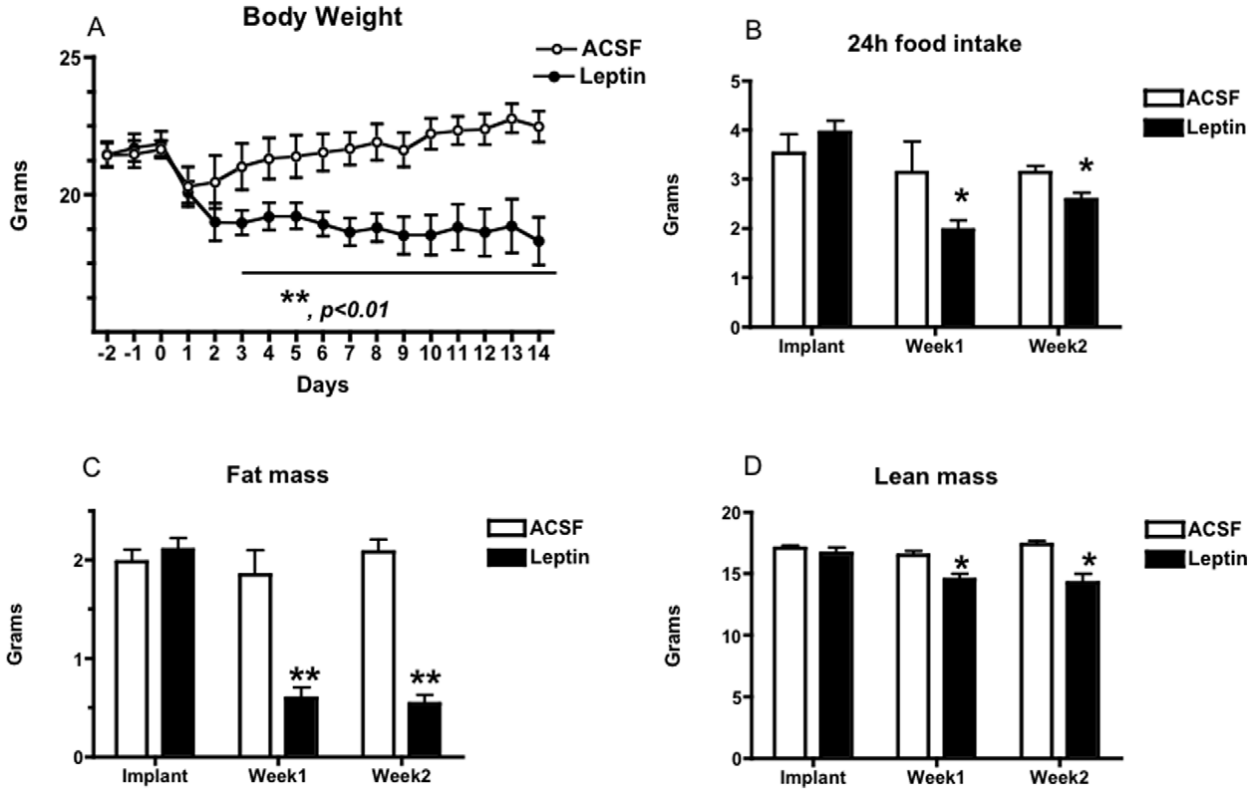
Body Weight, Food Intake, And Body Composition In Leptin Treated MKR Mice. Two week third intracerebroventricular leptin treatment (filled symbols) reduced body weight gain (A), 24 h daily food intake (B), fat mass (C) and produced a small reduction in lean mass (D) in male MKR mice, relative to artificial cerebrospinal fluid (ACSF) vehicle treated controls (open symbols). All data presented as means ± SEM, N = 6/group. * p<0.05, ** p<0.01.

**Figure 2:**
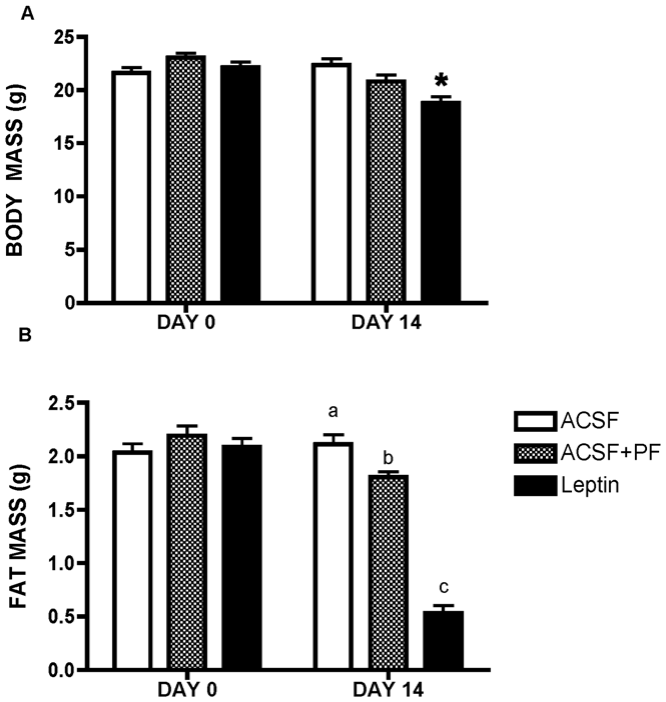
Body Weights (A) And Fat Content (B), Before And After 14 Days Of ICV Leptin Treatment Compared With ACSF And Pair-Fed Control Mice. All data presented as means ± SEM, N = 6–7/group. * p<0.05. Different superscript letters indicate significant differences at p<0.05.

Chronic ICV leptin infusion caused a ∼10–15% reduction in body weight (BW) in MKR mice that began within 1–2 days of infusion onset, and remained stable thereafter (Figure 1A), while ACSF was without effect. This reduction in body weight was accompanied at weeks 1 and 2 by significantly reduced food intake and fat mass, a small but significant reduction in lean mass (Figure 1B–D). Body weight and fat mass were also measured in pair-fed controls (Figure 2 A and B) and only leptin treated mice showed a marked decrease in fat mass. A small but significant reduction in circulating plasma leptin levels (1.7±0.2 ng/ml versus 2.0±0.3 ng/ml, p<0.05) was seen. Central leptin treatment also significantly reduced serum insulin, FFA and TG levels relative to ACSF treated controls (insulin: 1.2±0.23 vs. 3.2±0.3 ng/ml; FFA: 0.35±08 vs 0.75±12 ng/dl, p<0.01; TG: 0.2±0.1 vs. 1.4±0.3 nM (all p<0.01)).

**Figure 3:**
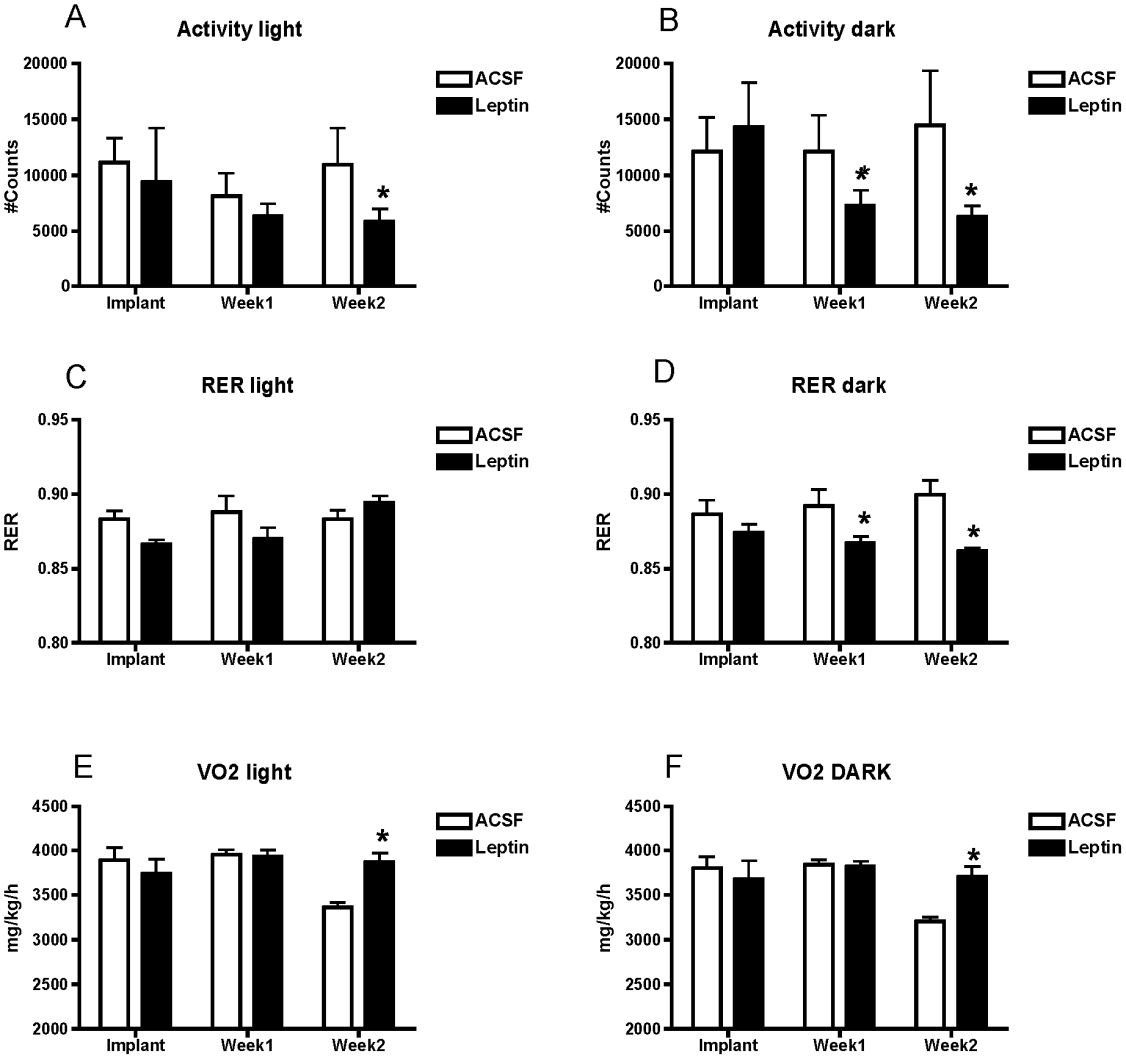
Locomotor Activity And Metabolic Functions In Leptin Treated MKR Mice. Two week third intracerebroventricular leptin treatment (black bars) reduced light and dark phase locomotor activity (A,B), respiratory quotient (RER, C,D) and increased both light and dark phase oxygen consumption in male MKR mice relative to artificial cerebrospinal fluid (ACSF) vehicle treated controls (open bars) (E,F). All data presented as means ± SEM, N = 6/group. * p<0.05.

Spontaneous locomotor activity during both light and dark phases was significantly reduced in ICV leptin treated MKR mice relative to controls (Figure 3A–B). Leptin also decreased respiratory equivalent ratio (RER) during the dark but not light phase, demonstrating increased fat oxidation (Figure 3C–D). These changes were accompanied by increased oxygen consumption during both light and dark phases in leptin treated mice (Figure 3E–F).

**Figure 4:**
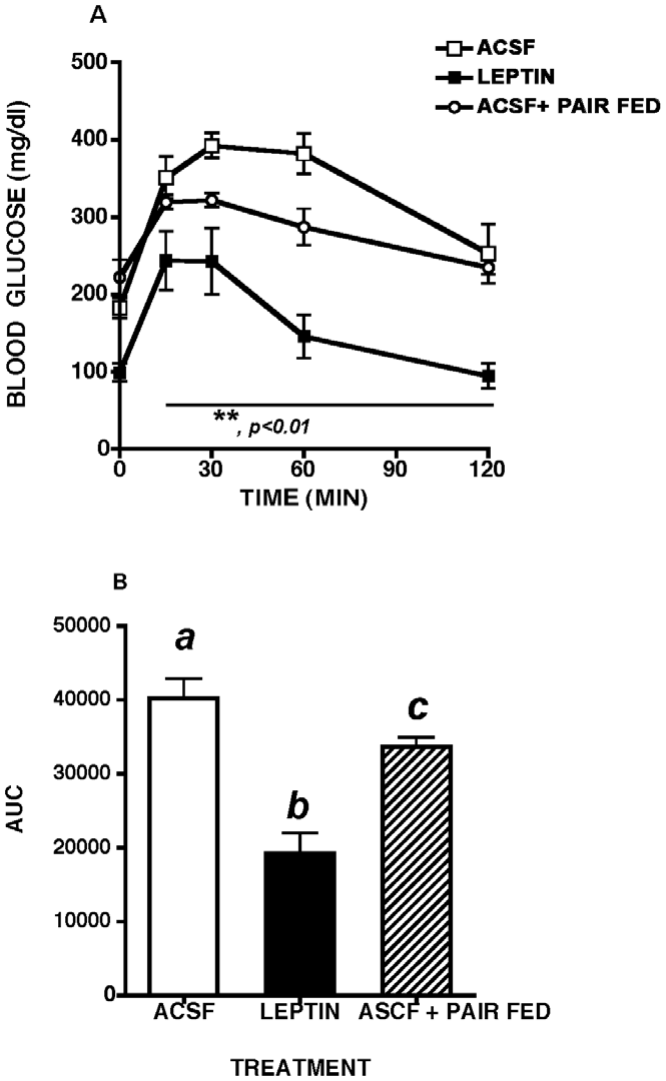
Glucose Tolerance Tests In Leptin And Vehicle Treated MKR Mice. Two week intracerebroventricular leptin treatment significantly improved glucose tolerance in male MKR mice relative to artificial cerebrospinal fluid (ACSF) vehicle treated controls and pair fed, ACSF treated MKR mice. A. Intraperitoneal glucose tolerance test (ipGTT, 2 g/kg), B. Area under the curve (AUC) during ipGTT. All data presented as means ± SEM, N = 6–7/group. * p<0.05, ** p<0.01.

Prior to and immediately following implantation of the infusion pumps, fed blood glucose levels remained in the diabetic range in all MKR mice (between 300–400 mg/dl). ICV leptin significantly lowered fasting blood glucose (BG) beginning 1 week following leptin infusion (98.8±14 mg/dl vs. 222±23 mg/dl, p<0.001), while pair feeding ACSF treated mice to the level of leptin treated controls was without effect (ACSF: BG 222±23 vs. ACSF pair fed: BG 182±13 mg/dl, N.S.). ICV leptin also significantly improved glucose tolerance relative to both ASCF and ASCF-pair fed controls (Figure 4A–B).

### Hyperinsulinemic Euglycemic Clamp Studies

**Figure 5:**
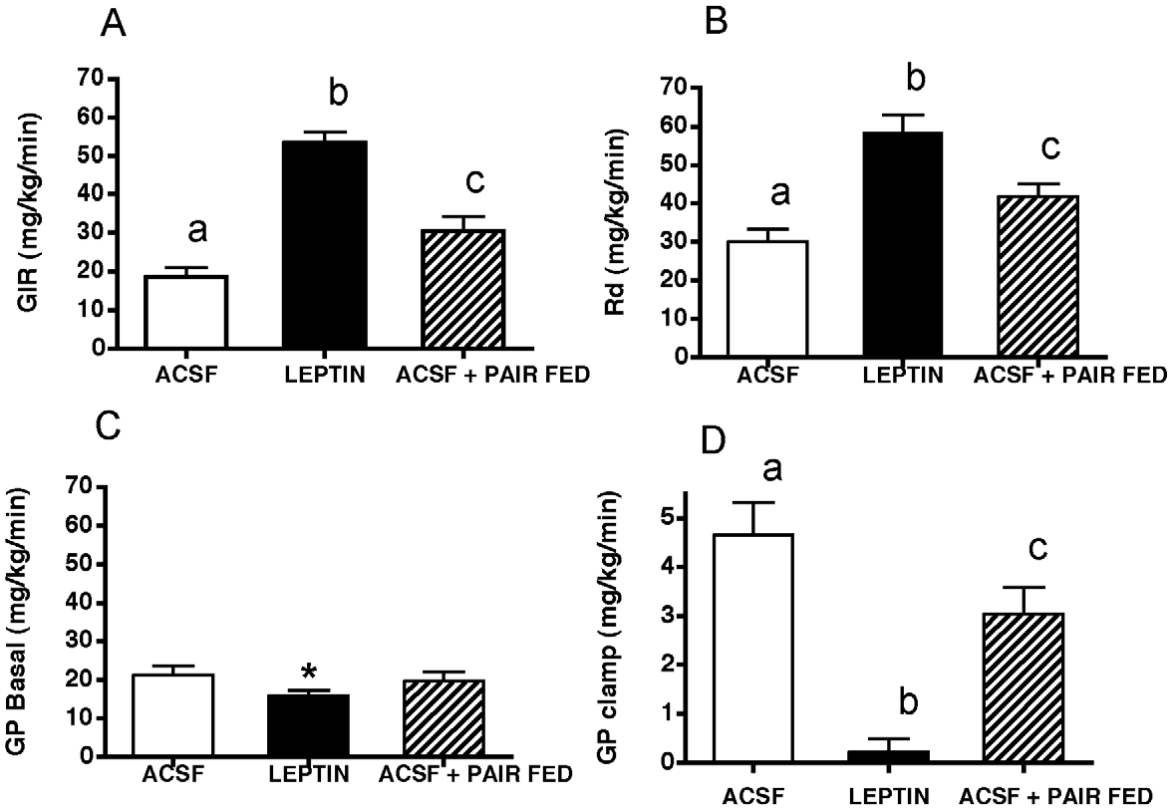
Hyperinsulinemic-euglycemic clamps. Third intracerebroventricular leptin treatment (black bars) significantly improved hepatic insulin sensitivity and glucose disposal during hyperinsulinemic-euglycemic clamps in male MKR mice relative to artificial cerebrospinal fluid (ACSF) vehicle treated controls (open bars) and pair fed, ACSF treated male MKR mice (hatched bars). A. Glucose infusion rate (GIR) during the clamp, B. peripheral glucose disappearance rate (Rd), hepatic glucose production (GP) under basal conditions (C) or hyperinsulinemia (D). All data presented as means ± SEM. N = 5–6/group. Different superscript letters indicate significant differences at p<0.05.

Glucose infusion rate (GIR) and the rate of glucose disappearance (Rd) during hyperinsulinemic-euglycemic clamps were significantly increased in central leptin treated-MKR mice relative to ACSF and ACSF, pair fed mice (Figure 5 A–B). Leptin also produced a small but significant reduction in basal glucose production (Figure 5C). However, endogenous glucose production during the clamp was greatly reduced in mice receiving central leptin infusion relative to both control groups (Figure 5C). Pair feeding alone significantly increased GIR and Rd and decreased glucose production during the clamps, although not to the degree elicited by central leptin infusions (Figure 5 A,B,D).

### Effects Of Hepatic Branch Vagotomy

**Figure 6:**
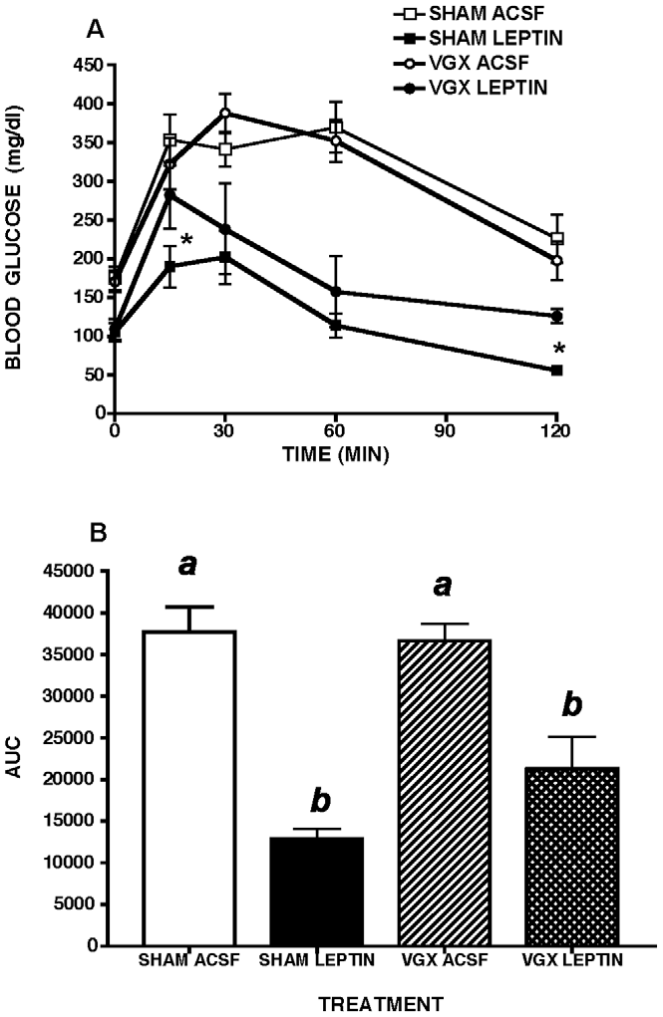
Glucose tolerance in leptin-treated MKR mice with or without hepatic vagotomy. Selective hepatic branch vagotomy (VGX) partially reversed the ability of two week third intracerebroventricular leptin treatment to improve glucose tolerance in male MKR mice, relative to artificial cerebrospinal fluid (ACSF) vehicle treated controls. SHAM  =  sham surgical treatment. A. Intraperitoneal glucose tolerance (ipGTT, 2 g/kg), * p<0.05, B. Area under the curve (AUC) during ipGTT. All data presented as means ± SEM, N = 6–7/group. Different superscript letters indicate significant differences at p< 0.05. * p<0.05.

Five hr. fasted blood glucose levels were lower in all leptin treated MKR mice compared to ACSF and ACSF pair fed controls, but this leptin-induced reduction was unaffected by hepatic branch vagotomy (LEPTIN + SHAM: 104 ng/dl ±18 vs. LEPTIN ± VAGOTOMY: 109 mg/dl ±13) (Figure 6A). Following ip glucose injection, hepatic branch vagotomized MKR mice showed significantly less of a reduction in blood glucose levels at 15 and 120 min, but their AUC for blood glucose did not differ from that of sham vagotomized mice infused with leptin (Figure 6A,B). Hepatic branch vagotomy alone failed to alter ipGTT in ACSF treated mice relative to sham surgical controls (Figure 6A,B).

## Discussion

The present results demonstrate that central leptin improves glucose homeostasis in the non-obese Type 2 diabetic MKR mouse. Third intracerebroventricular administration of leptin markedly increased whole body glucose tolerance as well as glucose disposal during hyperinsulinemic euglycemic clamps. We conjectured that brain leptin acted via the liver rather than the skeletal muscle to produce these effects, because muscle insulin- and IGF-1- induced glucose uptake into muscle is abrogated by the genetically engineered dnIGF-1R in MKR muscle [1]). Consistent with this idea, intracerebroventricular and hypothalamic leptin administration alters hepatic glucose fluxes and glucose production [6], [7], and such alterations are mediated in part via the intact hepatic vagus nerve [10]. Consequently, we evaluated the degree to which hepatic branch vagotomy affected the ability of central leptin to improve glucose homeostasis. Vagotomy modestly reversed the improvement in glucose tolerance in central leptin treated animals, but the overall area under the curve was not significantly changed, suggesting that the consequences of leptin's actions on other tissues may be playing a more important role in determining this effect. One possible mechanism is the reduction of substrates reaching the liver, such as fatty acids.

Accordingly, we report that adiposity, plasma TG and FFA were decreased and fat oxidation and oxygen consumption were increased in leptin treated animals relative to ACSF vehicle treated controls. These data suggest a direct neural action of leptin on lipid metabolism that is consistent with results from previous studies. Leptin activates sympathetic nerves supplying white adipose tissue via a central neural pathway to increase plasma glycerol and free fatty acid levels [12]. Furthermore, mediobasal hypothalamic administration of leptin decreases white adipose tissue (WAT) lipogenesis, and this effect is blocked by sympathetic denervation, supporting the existence of a hypothalamic WAT circuit mediating lipid metabolism [5]. In MKR mice, increased β3 adrenergic stimulation, targeting adipose tissue, improves whole body glucose homeostasis and hepatic insulin sensitivity, accompanied by reduced adiposity and increased fat oxidation [3]. Here we report that central leptin also improves hepatic glucose production and hepatic insulin sensitivity during clamps, again in parallel with reduced adiposity and increased fat oxidation. Thus, some of the present improvement in overall glucose homeostasis we observed may be secondary to leptin-induced reductions in adiposity in white adipose tissue, as supported by the inability to identify and retrieve epididymal, inguinal or omental white adipose tissue in animals after leptin treatment.

The increases in fat oxidation and reductions in plasma free fatty acids and TG following central leptin administration were accompanied by an increase in oxygen consumption. This occurred in spite of a significant reduction in total locomotor activity. The effect of ICV leptin on locomotor activity in the MKR mice contrasts the effect seen in other mouse models [13] and maybe specific to the MKR mice. Future studies should include wild-type control mice using the same FVBn background to address this discrepancy. On the other hand, part of the apparent discrepancy between increased oxygen consumption and reduced locomoter activity following ICV leptin, may be due to the ability of central leptin to increase sympathetic outflow to brown adipose tissue, thereby increasing thermogenesis [14]. Alternatively, skeletal muscle may be a metabolic target of central leptin action in the MKR mouse. Consistent with this suggestion, we found that hyperinsulinemic-euglycemic clamps in MKR mice receiving central leptin displayed a marked increase in the rate of disappearance of glucose. Intracerebroventricular and parenchymal hypothalamic administration of leptin have been demonstrated to increase skeletal muscle glucose metabolism, glucose uptake and fat oxidation, and these effects are blocked by either surgical denervation or by application of chemical agents that block sympathetic neural outflow to skeletal muscle [8], [9], [15], [16], [17]. These effects of central leptin appear to be mediated by brain melanocortin receptors, as central administration of the melanocortin 3/4 receptor antagonist SHU9119 blocks the ability of ventromedial hypothalamic (VMH) leptin to increase skeletal muscle glucose uptake, while ICV or direct VMH injection of melanoocortin receptor agonists increase uptake [18]. Since the present study did not include experiments to test these effects directly, our conclusions must remain speculative and await confirmation in future studies.

Interestingly, recent studies have demonstrated an effect of leptin in STZ-induced Type 1 diabetic rodents [19], [20]. Both peripheral and ICV administered leptin improved the hyperglycemic state, despite the absence of circulating insulin. These data as well as those from the present study, strongly suggest that the effects of leptin (whether via the hypothalamus or directly on peripheral tissues) on glucose homeostasis are unique and require further investigation.

In summary, we have shown that central leptin is an effective anti-diabetic therapy in a non-obese model of type 2 diabetes. Current evidence supports the suggestion that its beneficial metabolic effects in this model rely on distinct neural linkages between central leptin receptors and adipose tissue, liver and skeletal muscle.

“All animal protocols were approved by the IACUC committees of the Mount Sinai School of Medicine and the Albert Einstein College of Medicine of Yeshiva University.” Protocol Einstein #20090402 approval date 7/13/2009. Protocol Mt Sinai # IACUC MKR metabolism 05-1274.
